# Cyclomodulins and Hemolysis in *E. coli* as Potential Low-Cost Non-Invasive Biomarkers for Colorectal Cancer Screening

**DOI:** 10.3390/life11111165

**Published:** 2021-10-31

**Authors:** Kristýna Mezerová, Lubomír Starý, Pavel Zbořil, Ivo Klementa, Martin Stašek, Petr Špička, Pavel Skalický, Vladislav Raclavský

**Affiliations:** 1 Department of Microbiology, Faculty of Medicine & Dentistry, Palacký University Olomouc, Hněvotínská 3, 775 15 Olomouc, Czech Republic; vladislav.raclavsky@upol.cz; 2 First Department of Surgery, University Hospital Olomouc, I. P. Pavlova 6, 779 00 Olomouc, Czech Republic; lubomir.stary@fnol.cz (L.S.); pavel.zboril@fnol.cz (P.Z.); ivo.klementa@fnol.cz (I.K.); martin.stasek@fnol.cz (M.S.); petr.spicka@fnol.cz (P.Š.); pavel.skalicky@fnol.cz (P.S.)

**Keywords:** colorectal cancer, screening, genotoxin, colibactin, cytotoxic necrotizing factor

## Abstract

The frequent occurrence of *E. coli* positive for cyclomodulins such as colibactin (CLB), the cytotoxic necrotizing factor (CNF), and the cytolethal distending factor (CDT) in colorectal cancer (CRC) patients published so far provides the opportunity to use them as CRC screening markers. We examined the practicability and performance of a low-cost detection approach that relied on culture followed by simplified DNA extraction and PCR in *E. coli* isolates recovered from 130 CRC patients and 111 controls. Our results showed a statistically significant association between CRC and the presence of colibactin genes *clbB* and *clbN*, the *cnf* gene, and newly, the hemolytic phenotype of *E. coli* isolates. We also observed a significant increase in the mean number of morphologically distinct *E. coli* isolates per patient in the CRC cohort compared to controls, indicating that the cyclomodulin-producing *E. coli* strains may represent potentially preventable harmful newcomers in CRC patients. A colibactin gene assay showed the highest detection rate (45.4%), and males would benefit from the screening more than females. However, because of the high number of false positives, practical use of this marker must be explored. In our opinion, it may serve as an auxiliary marker to increase the specificity and/or sensitivity of the well-established fecal immunochemical test (FIT) in CRC screening.

## 1. Introduction

Bacteria and their metabolites are an integral part of the human intestine, maintaining a complex and working biosystem. However, intestinal microbiota are also connected to various gastrointestinal disorders, even to serious digestive tract diseases including sporadic colorectal cancer (CRC). The processes leading to the mutagenesis of oncogenes and tumor suppressor genes of intestinal mucosa have been associated with the bacterial microbiota composition in many studies [1,2,3]. There are a number of mechanisms through which bacteria can contribute to the formation of tumors [4]. One of them is the activity of cyclomodulins produced by certain microorganisms, *E. coli* in particular, while another is the toxigenic action of certain *Bacteroides fragilis* strains [5,6]. In addition, several associations of bacterial species with CRC or adenoma have been observed without understanding the background of such associations. These include individual species such as *Fusobacterium* spp., *Peptostreptococcus* spp., *Parvimonas,* or *Porphyromonas* spp. [5,7,8,9], complex bacterial signatures [10,11,12,13], or even newly described species (*Lachnoclostridium* sp. [14]).

In this study, we aimed to explore the possibility of low-cost culture-based detection of a potential bacterial CRC auxiliary marker suitable to improve the performance of current CRC screening strategies. With respect to this goal, we decided to explore the potential of cyclomodulin-producing *E. coli* because (i) *E. coli* is abundantly present in the stools of most people, (ii) it is easy to culture, and (iii) its DNA can be easily extracted from colonies and tested for the presence of cyclomodulin genes. To the best of our knowledge, all clinical studies relying on non-invasive sampling in pre-colonoscopy settings have thus far been based on solid stool sampling followed by DNA extraction and PCR [15]. Due to the presence of high amounts of human DNA, non-*E. coli* bacterial DNA, and potential PCR inhibitors in this type of sample, costly DNA extraction is required to achieve a satisfactory quality of template DNA. In addition, our strategy allows the use of a simple rectal swab for sampling, which can be performed at any time without the need to wait for the moment of discharge of feces from the body and to catch and handle the feces. Furthermore, a rectal swab culture can be performed within the standard workflow in any diagnostic microbiology laboratory. Then, such screening could be offered to all eligible patients not only as a planned solitary CRC screening test, but also on other occasions when rectal swabs are taken for any CRC-unrelated conditions. This may allow broad-range high-throughput screening, with the only extra effort required concerning obtaining informed consent.

### 1.1. Roles of Cyclomodulins

Cyclomodulins are able to modulate differentiation, proliferation, and apoptosis in eukaryotic cells, which could directly impact carcinogenesis via the activation of mutated pathways leading to tumor formation [16]. Three types of such molecules have been described in *E. coli* thus far—the colibactin, cytotoxic necrotizing factor, and cytolethal distending toxin.

#### 1.1.1. Colibactin

The genotoxin colibactin has been described as an inducer of DNA double-strand breaks (DSBs) in eukaryotic cells in vitro and in vivo leading to incomplete DNA repair, chromosomal aberrations, and cell cycle inhibition in the G2/M phase [17,18]. This genotoxin is spread by horizontal gene transfer, mostly among *E. coli* strains [19]. Colibactin is encoded on the 54 kb pks island, which carries *clb* genes for three non-ribosomal peptide synthases (NRPS: ClbH, ClbJ, ClbN), three polyketide-synthases (PKSs: ClbC, ClbI, ClbO), two hybrid synthases of the nonribosomal peptide synthase-polyketide synthase type (NRPS-PKS: ClbB, ClbK), and nine accessory proteins [17]. All 19 known *clb* genes are encoding products necessary for the regulation and activation of colibactin biosynthesis (namely clbA and clbR), transport, and even resistance (*clbS*) to colibactin itself [20,21]. Although studied since its discovery in 2006, the instability of the isolated toxin impeded disclosure of its complete structure until a study by Xue et al. in 2019, which was crucial for understanding its genotoxic mode of action. Pure samples of metabolites obtained from colibactin-producing *E. coli* allowed for the characterization of colibactin-DNA cross-links [22], while another study revealed a mature colibactin compound named colibactin-645 as the true effector molecule that induces DSBs in DNA via the copper-mediated oxidative mechanism in vitro [23] through DNA alkylation and interstrand crosslinking, with an increased probability to create mutations if unrepaired [24]. Unstable interstrand crosslinks undergo depurination followed by a slow rate of 3’-phosphate elimination, which could contribute to simultaneous DSB formation [25]. 

Genotoxicity resulting in potential pro-oncogenic activity of colibactin was suggested in many studies [18,26,27]. Cuevas-Ramos et al. described the induction of a transient DNA damage response in mammalian epithelial cells infected by *E. coli* harboring pks. The following cell division was accompanied by incomplete DNA repair, resulting in anaphase bridges and chromosome aberrations; infected cells exhibited increased frequency of gene mutations, indicating pro-oncogenic activity of colibactin [18]. Moreover, Dalmasso et al. demonstrated that transient contact of a few malignant cells with colibactin-producing *E. coli* results in the production of growth factors, which promote the proliferation of uninfected cells and hence drive them to tumor growth [26]. Further, a survey of somatic mutations at colibactin target sites of several thousand cancer genomes revealed the notable enrichment of a colibactin-specific DNA-damage signature motif in CRC [27]. Another recent study described a mutational signature in CRC human organoids as a consequence of single -base substitutions and deletions, which resulted from previous colibactin exposure [28]. Finally, the transformation of healthy epithelial cells to a premalignant state upon short-term exposure to colibactin-producing pks+ *E. coli* was demonstrated in vitro by generating cells that grew independently of the Wingless/Integrated (Wnt) signaling pathway. This pathway is strongly associated with colon tumors, and aberrant Wnt signaling was observed in more than 94% of CRC cases [29,30,31]. Moreover, pks+ *E. coli* was detected more frequently in CRC patients than in healthy controls [8,32].

#### 1.1.2. Cytotoxic Necrotizing Factor

Other cyclomodulins with pro-oncogenic activity such as the cytotoxic necrotizing factor (CNF) and the cytolethal distending toxin (CDT) are also associated with CRC [17,18]. CNF is mostly produced by uropathogenic *E. coli* (UPEC) as well as by diarrhea-causing *E. coli*. However, this cyclomodulin was also detected in *E. coli* isolated from patients with neonatal meningitis and oncological patients with bacteremia [33,34,35,36,37]. CNF is a cyclomodulin interfering with cytokinesis leading to morphological changes such as membrane ruffles, flattening of the cell body, and the formation of enlarged multinucleated cells [38]. Toxic and cell-altering effects were observed in the infected cells, which is in accordance with in vitro studies that demonstrate the ability of CNF to alter the cytoskeleton of cultured cells [39,40]. The morphological changes observed in infected cells are caused by the permanent activation of Rho GTPases, which participate in the arrangement of actin stress fibers [41]. All of these effects could contribute to malignant transformation. Human colon cancer cells exposed to CNF in vitro showed blockage of cytokinesis, endoreplication, and polyploidization, resulting in reversible senescence and thus genomic instability [42]. 

#### 1.1.3. Cytolethal Distending Toxin

Compared to other cyclomodulins, CDT is less frequent in CRC patients. Some *E. coli* isolates can produce CDT concurrently with colibactin or CNF [43,44,45]. Similar to colibactin, CDT is able to directly induce DSBs [46]. Following the exposure of cells to CDT, DNA single-strand breaks (SSBs) appear, which are subsequently converted to DSBs in the S phase [47], resulting in cell cycle arrest in the G2/M phase followed by cell apoptosis [48,49,50]. However, the formation of DSBs also leads to the activation of DNA damage responses, avoiding replication and thus resulting in genetic instability that favors tumor promotion in both proliferating and non-proliferating cells [51,52,53]. Another recent study demonstrated an increased number of tumors in mice infected by CDT+ Campylobacter jejuni. The genotoxic activity of CDT was also observed in rat small intestine epithelial cells and in human colon cancer cell lines, including chromosomal aberrations, cell cycle disruption, and more frequent mutations [54,55]. However, normal human colon epithelial cells exposed to CDT-producing *E. coli* displayed only modest changes compared to those induced in precancerous derivatives, suggesting that CDT+ *E. coli* is not a potent CRC initiator, but could be a promoter of malignant transformation [56].

## 2. Materials and Methods

### 2.1. Patients and Clinical Material

Newly diagnosed CRC patients aged 50 years or older and seeking primary care at the First Department of Surgery, University Hospital Olomouc were recruited as cases, whereas patients aged 50+ and seeking care at the same department for non-CRC conditions were recruited as controls, both during the period from July 2015 to May 2019. To prevent possible bias presumed in some patient groups and conditions, the following exclusion criteria were applied to all participants: (i) A personal history of digestive cancer or inflammatory bowel disease, or a family history of CRC (first-degree relatives); (ii) antibiotic use within the 2 months before sampling; (iii) diarrhea or other symptoms of gastrointestinal infection within the 2 weeks before sampling; (iv) bowel-clearing within 1 week before sampling (typically ≥2 weeks in newly diagnosed CRC patients). Rectal swabs were collected using the Transystem™ 116C bacteriology transport swabs (COPAN Diagnostics Inc., Murrieta, CA, USA) and transported to the laboratory on the same day.

### 2.2. Samples Processing

Swabs were shaken in 200 µL of Brain Heart Infusion Broth with 10% serum (Oxoid CZ, s.r.o., Brno, Czech Republic) and inoculated onto Columbia blood agar (CBA) and MacConkey agar plates (Trios spol. s r.o., Praha, Czech Republic). The plates were incubated at 37 °C for 2 days. Each colonial morphotype presumptively identified as enterobacterium, based on characteristic colony appearance as evaluated by an experienced microbiologist, was subcultured on CBA at 37 °C for 16–24 h and identified by MALDI-ToF MS using the Microflex LT/SH instrument and Biotyper software (Bruker Corporation, Billerica, MA, USA) in accordance with the manufacturers’ instructions. Hemolysis was evaluated on the same plates in isolates identified as *E. coli*, which were subsequently all stored at −80 °C in a cryoprotectant until tested further.

### 2.3. Genetic Analysis of E. coli Isolates

A thermal lysis procedure was used for DNA isolation from all collected *E. coli* isolates. Briefly, one 24-h-old bacterial colony was harvested and suspended in 50 µL of sterile deionized water. The bacterial suspension was incubated at 90 °C with 440 rpm shaking for 10 min and subsequently spun down at 13 000× *g* for 2 min. The supernatant was stored as a thermal lysate at −20 °C until used. For the detection of cyclomodulin genes, 1 µL of the thermal lysate was added to 20 µL of the reaction mixture consisting of 1× qPCR SYTO-9 Master Mix (Top-Bio s.r.o., Vestec, Czech Republic) and 0.1 μM of the respective forward and reverse primer (see Table 1 for a list of primers). 

Two different primer pairs targeting independent genes present inside the pks genomic island were used to increase the reliability of detection of an intact pks island needed for the appropriate encoding of colibactin. PCR amplification was performed in the LightCycler 96 instrument (Roche, Basel, Switzerland) in accordance with protocols previously published for colibactin genes [57], the CNF encoding gene [58], the cdtB gene [59], and *E. coli* phylotyping [60]. Primers used for this PCR are listed in Table 1. High-resolution melting analysis was used for product detection with the following conditions: 95 °C for 60 s, 40 °C for 60 s, 65 °C for 1 s, followed by melting from 65 to 92 °C at a 2.2 °C/s ramp rate with 25 readings per 1 °C.

### 2.4. Statistical Analysis

Except for differences in the number of distinct colonial morphotypes that were evaluated using the Mann–Whitney U test, all other associations were evaluated using the chi-square test or Fisher’s exact test. The statistical package IBM SPSS Statistics version 22 and the MedCalc software were used to analyze the data. The significance level was at 5% (*p* < 0.05)

## 3. Results

### 3.1. E. coli Culture Results

A total of 263 patients aged 50–90 years were recruited to participate in the study (Supplementary Material Table “Overview patient data”); most rectal swab cultures yielded *E. coli* (*n* = 241; 91.6%). There was no significant difference in *E. coli* positivity rates between CRC patients and controls. In many cases, more than one morphologically distinct *E. coli* isolate was recovered from one participant; namely, 208 isolates were from 130 CRC patients and 152 isolates were from 111 controls, a total of 360 isolates to be further studied. Interestingly, CRC patients harbored significantly more morphologically distinct *E. coli* isolates compared to controls (*p* = 0.007, Mann–Whitney U test). For a detailed analysis of this difference, see Section 3.4 below. For a breakdown of the underlying conditions in controls. see Table 2.

### 3.2. Evaluation of the Representativeness of the CRC Cohort

**Figure 1:**
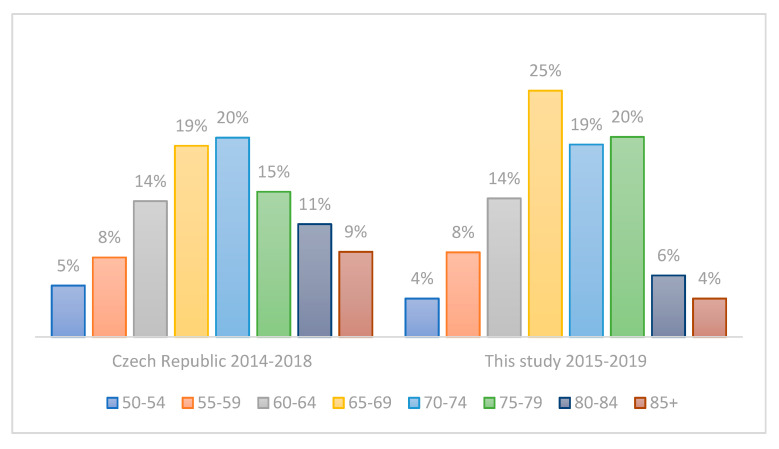
Distribution of CRC patients by age according to the Czech National Cancer Registry compared to CRC patients included in our study.

**Figure 2:**
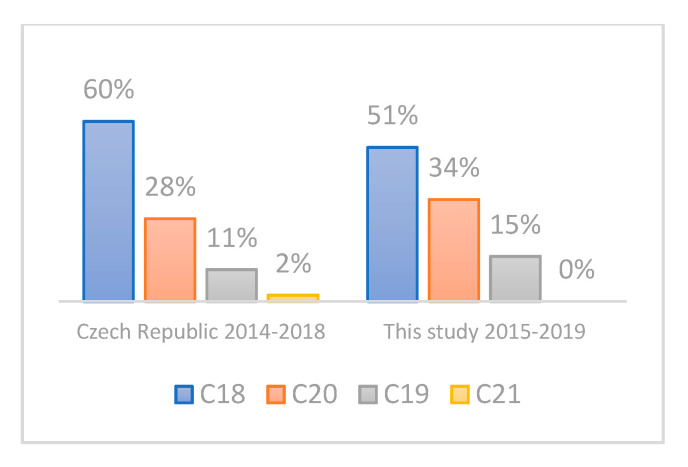
Distribution of CRC location in the Czech population aged 50+ in 2014–2018 according to the Czech National Cancer Registry compared to CRC patients included in our study 2015–2019. C18—malignant neoplasm of colon, C19—malignant neoplasm of rectosigmoid junction, C20—malignant neoplasm of rectum and C21—malignant neoplasm of anus and anal canal.

**Figure 3:**
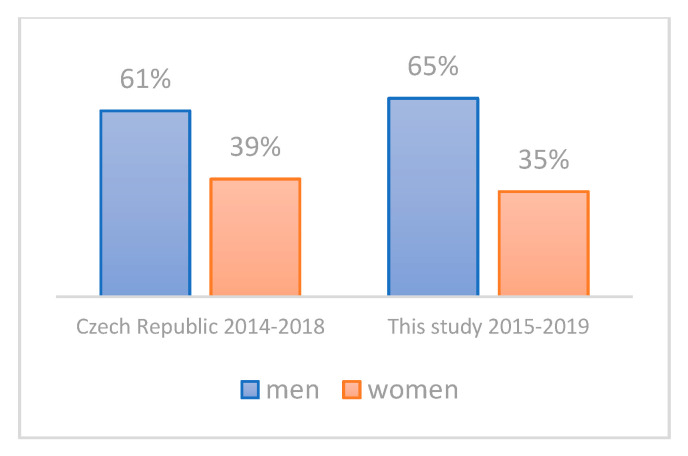
Sex distribution in CRC patients aged 50+. Bars show relative distribution among males (blue bars) and females (red bars). For the Czech population, relative percentages are based on cumulative incidence by sex in 2014–2018 according to the Czech National Cancer Registry. For this study, relative percentages are based on absolute numbers of patients included.

The main characteristics of the study cohort are summarized in Table 3. The mean ages of CRC patients and controls were 69.4 ± 8.15 and 68.6 ± 8.86, respectively, indicating the absence of any potential age-bias. The proportion of males in the CRC group was 66% (M/F: 93/47), compared to 50% in controls (M/F: 62/61); that is, there were 1.3 times more male CRC patients than male controls. This corresponds to the higher incidence of CRC in Czech males; according to the Czech National Cancer Registry, the mean incidence of CRC was 1.9 times higher in males aged 50+ compared to females in 2014–2018 (211.8 versus 108.4, respectively). Furthermore, there were no statistically significant differences in the age distribution (Figure 1) and tumor location distribution (Figure 2) between our group of CRC patients and a corresponding Czech CRC-positive population (age 50+, time period of 2014–2018) as retrieved from the Czech National Cancer Registry. The same was true for sex distribution in our CRC patients when comparing the cumulative incidence of CRC by sex in the Czech Republic in 2014–2018 according to the Czech National Cancer Registry (Figure 3).

### 3.3. Prevalence of Cyclomodulin Genes and Hemolysis and Implications for Usefulness in Screening

Results of the detection of cyclomodulin-coding genes, and the occurrence of the hemolytic phenotype that indicates the production of hemolysins, are summarized in Table 4. All colibactin-positive *E. coli* isolates were assigned to phylogroup B2 based on PCR detection of the *chuA* and *yjaA* genes and the DNA fragment *TspE4C2* [60]. In the case that a patient harbored more than one morphologically distinct *E. coli* isolate, all the isolates were examined separately, and cyclomodulin-coding genes or hemolysis were recorded as present in the patient, irrespective of whether they were detected in one or more of the patient’s distinct isolates. The putative CRC detection rate was calculated as the percentage of CRC patients that would be correctly identified as having the disease based on the positive result of a particular test. Because of the character of our study, we could not establish how long the patients had been positive with any of the tests prior to the diagnosis of their CRC status. Therefore, this putative detection rate represents the maximum detection rate that might be achieved in real life; the true values may be lower. Except for the cytolethal distending toxin gene, all the others (colibactin gene, cytotoxic necrotizing factor gene, and hemolysis) were found to be significantly associated with CRC. We also evaluated the significance and putative CRC detection rate of the combined detection of two or more potential markers (Table 4). 

Unfortunately, the increase in detection rate was rather limited. Apparently, the non-colibactin cyclomodulins and hemolysins were rarely present in colibactin-negative participants, or, in other words, they were mainly present in colibactin-positive participants. This is not surprising for the association of hemolytic phenotype and cnf-positivity, because of the tight linkage between cnf1 and *hlyA* genes. In addition, we explored the association of clb-positivity and hemolysis and clb-positivity and cnf-positivity. Both hemolysis and cnf-positivity were observed much more frequently in clb-positive isolates compared to those clb-negative (9.2× more for hemolysis and 21.5× more for cnf-positivity, both *p* < 0.001). All of our *E. coli* clb+ isolates were also phylotyped by triplex PCR assigning all of them to the B2 phylogroup, which is characterized as the most virulent one.

Surprisingly, although we observed a strong link between the hemolytic phenotype and cnf-positivity, not all of the cnf+ *E. coli* isolates were also hemolytic, which is in conflict with the tight linkage between cnf1 and *hlyA* genes. Because we followed the hemolytic phenotype, not the presence of *hlyA* in the genotype, lack of *hlyA* expression may be the most plausible explanation of this observation. Finally, to be sure about the robustness of our data, we also looked at the variability of the colibactin detection rate in CRC and control patients during the time of the study. This should unveil any potential bias that might influence the data in case the quality of testing varied during the time of the study. Neither unusual fluctuations nor significant differences in year-on-year comparisons were observed (Supplementary Material Figure “Colibactin detection rate in CRC and control patients for each year of the study”). 

### 3.4. Deciphering the Different Burden of Distinct E. coli Isolates in CRC Patient versus Controls

**Figure 4:**
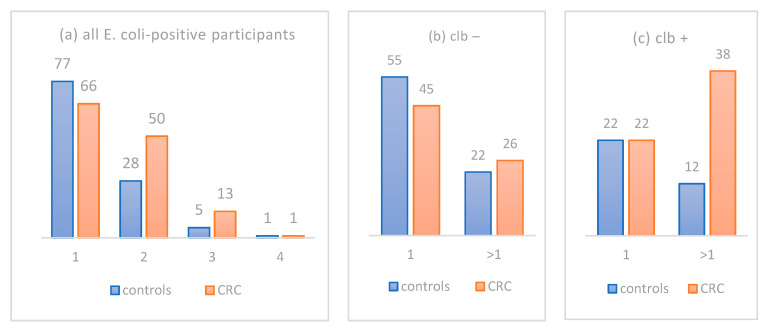
Different burdens of distinct *E. coli* isolates with respect to CRC status and colibactin status. X axis represents number of distinct *E. coli* isolates; Y axis represents number of participants; (**a**) burden of all *E. coli* isolates irrespective of their colibactin status; (**b**) burden of colibactin-negative, and (**c**) colibactin-positive isolates.

As already stated, CRC patients harbored significantly more morphologically distinct *E. coli* isolates compared to controls, namely 208 in 130 CRC patients (1.60 per patient) compared to 152 isolates in 111 controls (1.37 per patient; *p* = 0.007, Mann–Whitney U test). To gain better insight into this difference, we first plotted the counts of participants harboring different numbers of morphologically distinct *E. coli* isolates (Figure 4a) with respect to their status as controls (blue bars) or CRC patients (orange bars). Now, let us postulate that the distribution of isolates in the control group (blue bars) does represent the normal distribution in a healthy population. In such a case, Figure 4a clearly shows that the increased average number of *E. coli* isolates per patient in the CRC group (orange bars) mainly occurs as a result of the increased number of patients who carry two distinct isolates. Thus, a cyclomodulin-producing *E. coli* isolate might be the newly acquired “second” isolate added to the populational background of the “first” original harmless *E. coli* in CRC patients.

To explore whether we can find any support for this hypothesis, we counted the number of participants that carry either just one or more than one distinct *E. coli* isolate, separately in the group of colibactin-negative (Figure 4b) and -positive participants (Figure 4c). A remarkable difference was observed only when participants carried more than one distinct *E. coli* isolate and, at the same time, one of these isolates was colibactin-positive (Figure 4c, right). Among these 50 participants (clb+ and hosting >1 isolate), 38 were CRC patients and only 12 were controls, a 3.2:1 ratio, in contrast to a balanced 1:1 ratio in those participants who hosted just one isolate, making the difference highly significant (*p* = 0.016). Next, we looked at the male-to-female ratio in that deviating subgroup of 38 clb-positive CRC patients hosting >1 distinct *E. coli* isolate. Intriguingly, 28 of them were males and only 10 were females (2.8:1 ratio). In contrast, the corresponding group of 22 clb-positive CRC patients hosting just one distinct *E. coli* isolate consisted of 14 males and 8 females (1.8:1 ratio, i.e., almost identical to the 1.9:1 ratio for CRC incidence in the Czech population). Altogether, our data show that the presence of a colibactin-positive *E. coli* isolate most probably represents new colonization that is typically additional to previous colibactin-negative *E. coli* colonization. Furthermore, male sex seems to be associated with colibactin-positive *E. coli* colonization.

### 3.5. Evaluation of Potential Benefit of Toxin Screening in Different Age Groups, in Males versus Females, and in Different Disease Stages

Further remarkable differences were revealed by a detailed analysis of the data. When participants were divided into early old age (50–74 years old) and middle old age (75–90), detection of the potential CRC markers appeared to be markedly more beneficial in those aged 75–90 compared to those aged 50–74 years. Because colibactin turned out to be the most sensitive marker, we first summarize the data for colibactin alone and then for all the potential markers combined (Table 5). Similarly, detection of the markers also appeared to be more beneficial to males compared to females, irrespective of their age. In the end, when all combinations of age and sex were evaluated in relation to the potential benefit of colibactin screening, the results were surprisingly conflicting and will be discussed later. In males, testing would be more beneficial in middle old age (75–90; Table 6), whereas in females, the same was true in early old age (50–74; Table 7).

To reveal any relationship between *clb*+ *E. coli* status and TNM classification of the disease, we also evaluated the relative proportion of early versus advanced stages according to *clb*+ detection rate. Interestingly, a higher percentage of patients carrying clb+ *E. coli* were observed in T1/T2 (24/42 = 57%) compared to T3/T4 (30/88 = 34%; *p* = 0.012), in N0 (38/75 = 51%) compared to N ≥ 1 (25/51 = 49%; *p* = 0.018), and in M0 (45/98 = 46%) compared to M ≥ 1 (3/13 = 23%; *p* = 0.118). Because of the observational character of our study, with *E. coli* status being examined at the time of diagnosis only and not earlier or later, we could only speculate about the background of this difference. Nevertheless, it was found to be statistically significant in relation to both T and N staging. In addition, the difference in M staging showed the same, albeit not significant, tendency. The factors responsible for this difference remain a puzzle to us and should be explored in future studies. 

## 4. Discussion

Earlier studies that evaluated the usefulness of *E. coli* toxin gene detection as a potential marker of CRC relied on direct PCR detection of the respective genes in stool samples, which requires self-sampling of a solid stool sample and its delivery by the patient. Our approach may be advantageous because of the possibility to obtain a rectal swab sample directly and conveniently during a visit of any person eligible for CRC screening to any healthcare facility. Furthermore, techniques of subsequent *E. coli* culture from rectal swabs are widely available. Moreover, colonial DNA extraction and PCR amplification for the purpose of cyclomodulin gene detection also do not require special expertise and should therefore be robust for routine screening purposes.

It should also be noted that most of the studies published so far either did not consider the age of CRC patients and thus also included those younger than 50 years of age, or they did not specify the age of CRC patients at all. To best explore the usefulness of our approach for screening purposes, we focused on patients and controls aged 50–90 years, which is the population eligible for CRC screening in most countries. One limitation of this study is that the controls do not represent a truly healthy population. However, they were recruited among patients who sought care of the First Department of Surgery, University Hospital Olomouc, for non-CRC related and non-inflammatory conditions, thereby reducing the possible bias to a minimum (Table 1). In addition, the age, tumor location, and sex distribution in our cohort of CRC patients correspond very well with the respective parameters of the population of Czech CRC patients (Figure 1, Figure 2 and Figure 3). The focus on *E. coli* isolates solely might be another limitation of this study, because other enterobacterial species can harbor cyclomodulin genes as well [61]. In actuality, the fraction of clb-positive isolates non-*E. coli* isolates should, however, be very low in the general population, as described by Ballén V. et al. [62] recently, who detected *clb* in as few as 2 of 127 *K. pneumoniae* clinical isolates.

### 4.1. Colibactin Established as the Most Abundant Potential CRC Marker

Our results showed statistically significant associations between newly diagnosed CRC and the presence of clb-positive, cnf-positive, and hemolytic *E. coli* isolates in rectal swabs (Table 4), whereas no association was observed in the case of the cdt gene. These findings are in good agreement with previous studies [63,64]. The most abundant potential marker was the colibactin gene, which was found in 45.4% (59/130) of our *E. coli*-positive CRC patients. The cyclomodulin was detected in 55.3% (21/38) of CRC mucosal samples examined by Buc et al. [63], and in 56.4% (22/39) of stool samples from CRC patients examined by Eklöf et al. [8]. The higher detection rates of the colibactin gene observed by others highlight one possible limitation of our study, which is based on the subculture and further examination of typically one colony of each morphotype of *E. coli*. Therefore, in our samples, we cannot rule out the presence of more, different strains of *E. coli* showing the same morphology, where only some of them might harbor the colibactin gene and thus might be missed when subculturing and examining one representative colony only. Therefore, extensive examination of several colonies is recommended for future studies, possibly also accompanied by an examination of the strain diversity by some of the available molecular typing techniques. Furthermore, a reduced detection rate cannot be excluded if a less experienced technician were to perform the selection of colonies for subculture and MALDI ToF MS identification. However, presumptive identification of enterobacteria based on colonial morphology is one of the easier tasks in a routine diagnostic laboratory and can even be simplified when using selective media. In this study, a technician with >8 years of experience in the field performed this step. Another explanation of the lower colibactin detection rate achieved in our study might be a possibly higher yield because of direct sampling of the tumor mucosa in the first published study [63], and a likely higher sensitivity of qPCR examination of DNA extracted from solid stool samples in the second published study [8], compared to the recovery of *E. coli* from rectal swabs in our study. Therefore, we recommend enrichment of enterobacteria in selective broth prior to plating to increase the sensitivity in any future culture-based assays of cyclomodulins.

### 4.2. Other Cyclomodulin Genes Are Detected Less Frequently and Are Mostly Linked to the Presence of Colibactin

Compared to colibactin abundance, all the other potential markers studied by us occurred less frequently in CRC. However, it might be beneficial to combine colibactin detection with the detection of the other three less-sensitive markers to further increase the screening efficacy. Therefore, we explored this option (Table 4). The rather limited additive effect of combined detection indicated non-random co-occurrence of the potential markers, which was confirmed by statistical analysis. This finding can be explained by the plain fact that cyclomodulin-producing *E. coli* strains are dominantly recruited from the B2 phylogroup of *E. coli* [45,63,65], which increases the probability of sharing genetic traits. In our study, all colibactin-positive isolates belonged to the B2 phylogroup. Moreover, the hemolytic phenotype and the underlying *E. coli* hemolysin-coding gene *hlyA* have recently been associated with CRC [66]. Our study is only the second one that reports the association of this easily observable phenotypic trait with CRC. Nevertheless, based on our results, we recommend focusing mainly on colibactin detection when developing any screening assays in the future.

### 4.3. Males Should Benefit from Toxin Detection in CRC Screening More Than Women

To explore our data in detail, we also evaluated them in relation to sex and age groups of early old-aged (50–75 years old) and middle old-aged (75–90) participants (Table 5, Table 6 and Table 7). Although any such fragmentation of a cohort inevitably leads to a reduction of statistical power, it can point to tendencies worthy of exploration in the future. Interestingly, whereas there was no difference in the colibactin detection rate between early old-aged (50–74) men and women (44.1 and 43.8%, respectively), a puzzling difference was observed in those of middle old age (61.5% in men and 23.1% in women, respectively; *p* = 0.041). To the best of our knowledge, the only distinctive feature of older females versus males in CRC patients published so far is the progressive shift of the tumor location to the right side (proximal colon) with increasing age in women, indicating possible oncologic background differences in the elderly [67].

### 4.4. Cyclomodulin-Producing E. coli May Represent Harmful and Possibly Preventable Newcomers to Colon Microbiota

The significant difference between the number of morphologically distinct *E. coli* isolates between CRC patients (208 isolates from 130 patients, i.e., 1.6 isolate per patient) and controls (152 isolates from 111 patients, i.e., 1.35 isolate per patient; *p* = 0.007, Mann–Whitney U test) represents another new finding of this study. Intriguingly, increased occurrence of colibactin-positive isolates in CRC patients may be responsible for this difference. Let us presuppose that the frequency of colibactin-positive isolates observed in the control group (30.6%; 34/111) represents their baseline frequency in the population. In such a case, the same 30.6%, i.e., 40 of the 130 CRC patients, would represent the “healthy population colibactin-positive background” in the CRC group. Then, the additional 19 colibactin-positive isolates observed in the CRC group should represent CRC-related *clb+* newcomers compared to controls. The hypothesis regarding colibactin-positive *E. coli* as a harmful newcomer to the colon previously colonized by cyclomodulin-nonproducing *E. coli* is further supported by the analysis of multiple *E. coli* isolates in different subsets of our cohort as detailed in Section 3.4 in the Results and in Figure 4. Moreover, males seem to be predisposed to such colonization, which is in accordance with recent data by Watanabe et al. [68], who reported a positive association of pks+ *E. coli* with male sex (OR, 2.27 [95% CI 1.05–4.91]) in a healthy Japanese population. 

### 4.5. Open Questions and Perspectives

Obviously, our study cannot clarify whether the supernumerary colibactin-positive CRC patients are people who were healthy and later developed CRC because of the tumor-driving role of cyclomodulin-producing *E. coli*, or whether they are CRC patients who were excessively colonized by cyclomodulin-producing *E. coli* as passengers, because their CRC status favors such colonization, as proposed by Wassenaar [69]. Although unimportant when used as a biomarker, the search for potential sources of cyclomodulin-positive *E. coli* would be highly desirable in the case of their driving role. The very recent study by Watanabe et al. did not find any such source in diets [66], while Fabian et al. suggested small mammalian pets as a potential reservoir for cyclomodulin-producing *E. coli* [70].

Concerning the usefulness as a biomarker of CRC, the numbers of false positives and false negatives hinder the immediate practical usefulness of any colibactin assay. In our opinion, colibactin detection may, however, contribute to the performance of the well-established FIT as an auxiliary marker, namely in two ways. The first way would be analogous to that already outlined by Malagón et al., who demonstrated that a new bacterial signature for CRC screening reduced the false-positive rate of FIT in a screening population, promising to decrease the burden of unnecessary colonoscopies [12]. Secondly, anticipated by us when designing this study, colibactin-positive *E. coli* screening would be performed within the standard workflow of a diagnostic microbiology laboratory on *E. coli* colonies of any eligible patients (aged 50+) who gave informed consent on this testing when being sampled by rectal swab for any conditions. Of course, in such a setting, the patients must not be misled that undergoing CRC screening would replace the well-established FIT. However, they would not have to seek CRC screening actively, rather just give consent to an extended examination of their sample collected for other purposes. A possibly positive result should then increase their uptake of FIT or even indicate colonoscopy as the next step. In the Czech Republic, the uptake of CRC screening reached only 22.7% in 2010, according to the most recent published data [71], lagging far behind the acceptable (≥45%) and recommended (≥65%) levels according to EU guidelines. 

## 5. Conclusions

1. Our data confirm the association of clb+, cnf+ and hemolytic *E. coli* strains with CRC.
2. Cultures from rectal swab followed by colony PCR may represent a viable and economical alternative to non-culture detection of toxigenic *E. coli* in stool samples, provided that its sensitivity be successfully increased.
3. It needs to be established whether the detection of cyclomodulin-producing *E. coli* does increase the sensitivity of current non-invasive CRC screening strategies. Clinical trials that would encompass simultaneous FIT and cyclomodulin-producing *E. coli* detection in the general population are urgently needed.

## Figures and Tables

**Table 1:** Primers used in the study.

Target Gene	Primer Name	Primer Sequence	^1^L (bp)	^2^T_a_ (°C)	Reference
*clbB*	clbB—F	GAT TTG GAT ACT GGC GAT AAC CG	579	57	[57]
	clbB—R	CCA TTT CCC GTT TGA GCA CAC			
*clbN*	clbN—F	GTT TTG CTC GCC AGA TAG TCA TTC			
	clb N—R	CAG TTC GGG TAT GTG TGG AAG G			
*cnf1*	cnf 1—F	GGCGACAAATGCAGTATTGCTTGG	552	63	[58]
	cnf 1—R	GACGTTGGTTGCGGTAATTTTGGG			
*cdtB*	CDTs1—F	GAAAGTAAATGGAATATAAATGTCCG	466	56	[59]
	CDTas1—R	AAATCACCAAGAATCATCCAGTTA			
	CDTs2—F	GAAAATAAATGGAACACACATGTCCG			
	CDTas2—R	AAATCTCCTGCAATCATCCAGTTA			
*chuA*	chuA.1F	GACGAACCA ACGGTCAGGAT	279	60	[60]
	chuA.2R	TGCCGCCAGTACC AAAGACA			
*yjaA*	yjaA.1F	TGAAGTGTCAGGAGACGCT G	211		
	yjaA.2R	ATGGAGAATGCGTTCCTCAAC			
TspE4.C2	TspE4C2.1F	GAGTAATGTCGGGGCATTCA	152		
	TspE4C2.2R	CGCGCCAACAAAGTATTACG			

^1^ L—product size; ^2^ Ta—annealing temperature of primers.

**Table 2:** Distribution of controls by diagnosis.

	*n*	%
Hernia	47	42
Gallstones	33	30
Non-CRC malignancy	13	12
Hemorrhoids	10	9
Other	8	7

**Table 3:** Main characteristics of the study cohort.

	All Participants		
	All	CRC+	Controls
*n*	263	140	123
Males	155	93	62
Females	108	47	61
Mean age	69.1	69.4	68.6
SD	8.49	8.15	8.86
	***E. coli-*positive participants**		
*n*	241	130	111
isolates recovered	360	208	152
Males	141	86	55
isolates recovered	225	147	78
Females	100	44	56
isolates recovered	135	61	74
Mean age	69.2	69.5	68.9
SD	8.43	8.07	8.85

**Table 4:** Results of the detection of cyclomodulin-coding genes, occurrence of hemolytic phenotype, and evaluation of the potential of combined testing.

	CRC	Controls	*p*	Detection Rate (%)
*E. coli-*positive participants	130	111		
*clb*+	59	34	0.019	45.4
*cnf*+	39	19	0.020	30.3
*cdt*+	10	8	0.886	-
Hemolytic	45	23	0.017	34.6
Positive for at least one of the following				
*clb+*, *cnf+*	61	35	0.015	46.9
*clb+*, hemolytic	64	37	0.013	49.2
*clb+*, *cnf+*, hemolytic	64	37	0.013	49.2

**Table 5:** Evaluation of potential benefit of cyclomodulin screening in different age groups.

	CRC	Detection Rate (%)
*E. coli-*positive participants aged 50–74	91	
of those		
*clb*+	40	44.0
Positive for at least one of the following		
*clb+*, *cnf+*, hemolytic	44	48.3
*E. coli-*positive participants aged 75–90	39	
of those		
*clb*+	19	48.7
Positive for at least one of the following		
*clb+*, *cnf+*, hemolytic	20	51.3

**Table 6:** Evaluation of potential benefit of cyclomodulin screening in different age groups in males.

	CRC	Detection Rate (%)
*E. coli-*positive males aged 50–90	85	
of those		
*clb*+	42	49.4
Positive for at least one of the following		
*clb+*, *cnf+*, hemolytic	44	51.8
*E. coli-*positive males aged 50–74	59	
of those		
*clb*+	26	44.1
Positive for at least one of the following		
*clb+*, *cnf+*, hemolytic	28	47.5
*E. coli-*positive males aged 75–90	26	
of those		
*clb*+	16	61.5
Positive for at least one of the following		
*clb+*, *cnf+*, hemolytic	16	61.5

**Table 7:** Evaluation of potential benefit of cyclomodulin screening in different age groups in females.

	CRC	Detection Rate (%)
*E. coli-*positive females aged 50–90	45	
of those		
*clb*+	17	37.8
Positive for at least one of the following		
*clb+*, *cnf+*, hemolytic	20	44.4
*E. coli-*positive females aged 50–74	32	
of those		
*clb*+	14	43.8
Positive for at least one of the following		
*clb+*, *cnf+*, hemolytic	16	50.0
*E. coli-*positive females aged 75–90	13	
of those		
*clb*+	3	23.1
Positive for at least one of the following		
*clb+*, *cnf+*, hemolytic	4	30.8

## Data Availability

The data presented in this study are available on request from the corresponding author. The data are not publicly available due to the protection of privacy of patients included in the study.

## Supplementary Materials

The following are available online at https://www.mdpi.com/article/10.3390/life11111165/s1, Figure “Colibactin detection rate in CRC and control patients for each year of the study”, Table “Overview patient data”.
