# Supplementary material for: Cyclomodulins and Hemolysis in E. coli as Potential Low-Cost Non-Invasive Biomarkers for Colorectal Cancer Screening
Source: Life (Basel). 2021 Oct 31;11(11):1165. doi: 10.3390/life11111165 (PMC8621933; doi:10.3390/life11111165)
Supplement: Supplementary file 1 [file life-11-01165-s001.zip › Supplementary figure.pdf]

Supplementary figure.

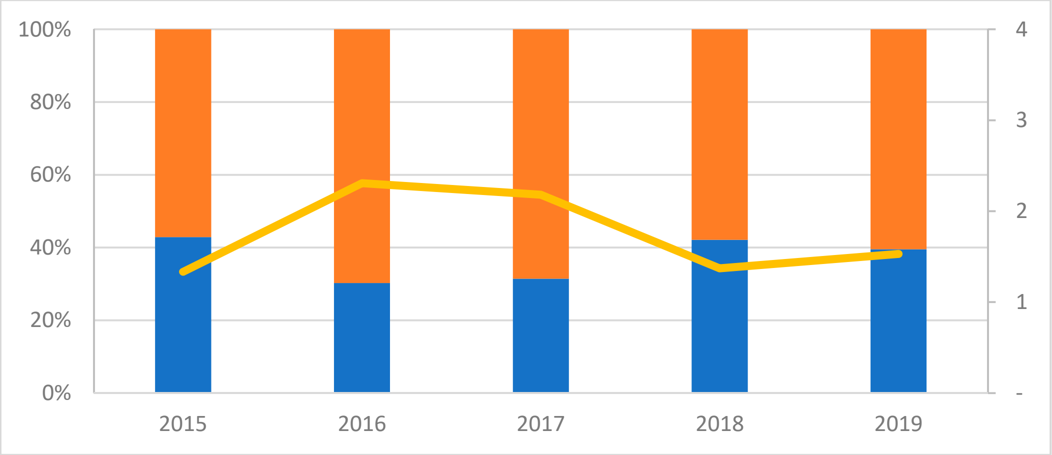

Figure S1. To unveil any potential bias that might influence the data in case the quality of testing would vary during the time of study, we evaluated the detection rate of colibactin genes clbB and clbN separately for the group of CRC patient and controls separately for each year of the study. Blue bars represent the relative (%) colibactin detection rate in controls, orange bars the same in CRC patients, whereas the yellow curve shows the variation in relation (index) between the detection rate in CRC versus controls. Namely, when the index would be 1 at the right Y axis, the ratio of detection rate in CRC versus controls would be 1 : 1 (i.e. 1/1 = 1). When this index is above 1, colibactin is detected more frequently in CRC compared to control. Obviously, there was no tendency of increasing colibactin detection rate during the study, that would indicate improved quality of testing during time. Also, there no unusual fluctuation in the index of detection rate of CRC versus controls. Also, statistical evaluation by chi square test did not show any significant difference when the data were compared thoroughly, i.e. all combinations of each year versus each year.
